# Reflections of students graduating from a transforming medical curriculum in South Africa: a qualitative study

**DOI:** 10.1186/1472-6920-12-49

**Published:** 2012-06-28

**Authors:** Lionel Patrick Green-Thompson, Patricia McInerney, Dianne Mary Manning, Ntsiki Mapukata-Sondzaba, Shalote Chipamaunga, Tlangelani Maswanganyi

**Affiliations:** 1 Centre for Health Science Education, Faculty of Health Sciences, University of the Witwatersrand, 7 York Rd, Parktown, Johannesburg, 2193, South Africa

## Abstract

**Background:**

The six year medical programme at the University of the Witwatersrand admits students into the programme through two routes – school entrants and graduate entrants. Graduates join the school entrants in the third year of study in a transformed curriculum called the Graduate Entry Medical Programme (GEMP). In years I and 2 of the GEMP, the curriculum is structured into system based blocks. Problem-based learning, using a three session format, is applied in these two years. The curriculum adopts a biopsychosocial approach to health care, which is implemented through spiral teaching and learning in four main themes – basic and clinical sciences, patient-doctor, community- doctor and personal and professional development. In 2010 this programme produced its fifth cohort of graduates.

**Methods:**

We undertook a qualitative, descriptive and contextual study to explore the graduating students’ perceptions of the programme. Interviews were conducted with a total of 35 participants who volunteered to participate in the study. The majority of the participants interviewed participated in focus group discussions. The interviews were transcribed verbatim and analysed thematically, using Tesch’s eight steps. Ethics approval for the study was obtained from the Human Research Ethics Committee of the University of the Witwatersrand. Participants provided written consent to participate in the interviews and for the interviews to be audio-taped.

**Results:**

Six themes were identified. These were: two separate programmes, problem-based learning and Garmins® (navigation system), see patients for real, being seen as doctors, assessment: of mice and MCQ’s, a cry for support and personal growth and pride. Participants were vocal in their reflections of experiences encountered during the programme and made several insightful suggestions for curriculum transformation. The findings suggest that graduates are exiting the programme confident and ready to begin their internships.

**Conclusions:**

The findings of this study have identified a number of areas which need attention in the curriculum. Specifically attention needs to be given to ensuring that assessment is standardized; student support structures and appropriate levels of teaching. The study demonstrated the value of qualitative methods in obtaining students’ perceptions of a curriculum.

## Background

The evaluation of an education programme is an essential component of the process of curriculum development and implementation [1]. Undergraduate medical programmes are routinely evaluated by students at various points during their training [2,3] and the use of questionnaires for graduates several years after graduation has been reported to provide useful data on how well prepared students have been for practice [4-6]. Watmough *et al.*[7,8] conducted interviews with graduates of both old and new curricula to compare perceptions of preparedness for practice. While data collected some time after graduation provides information derived from experience as practitioners, questioning students at the time of graduation is potentially valuable as their impressions of their experiences as students will not have been contaminated or diluted by post-graduation experiences. The AAMC [9] sends questionnaires annually to graduating medical students in the USA, but there do not appear to be any studies which report the use of interviews with medical students at the point of completion of their undergraduate studies. Interviews have the advantage over questionnaires of providing the richness and depth which is the hallmark of qualitative studies [10].

The final four years of the six-year medical degree programme at the University of the Witwatersrand, Johannesburg was comprehensively transformed in 2003. At the same time graduate entry was introduced to the degree which had traditionally only admitted students from high school into the first year of study. Graduate entry provides access to students from previously disadvantaged demographic groups who had not met the entry requirements immediately after high school as well as to school leavers who had been undecided on a career path [11]. Graduates also bring maturity, motivation and a wider range of life experience and diversity which should enhance the learning experiences of the entire cohort.

The new admission policy allows the graduates to join the school entrants in the third year of study. From that point the two groups follow an identical programme in a single track and years three to six of the medical degree are referred to as the Graduate Entry Medical Programme (GEMP). Years one and two of the GEMP are thus equivalent to years three and four of the degree and this part of the curriculum comprises organ system based blocks which largely focus on the basic sciences and the basic pathology disciplines. Problem based learning (PBL) is used in these two years using a three session format. In the first PBL session (PBL 1) students are introduced to the problem of the week through an audio-visual trigger. Working in groups of 10–12 students, with a trained facilitator who is either a clinician or a scientist students use a six-stage process to analyse the problem. This process begins with the audio-visual presentation, followed by an analysis of the problem, including clarification of terms. A biopsychosocial approach is used to analyse the problem. Students are then expected to formulate a problem statement and develop hypotheses about the mechanisms underlying the problem. Finally the hypotheses are ranked and learning objectives are identified. Learning objectives are developed around four central themes - basic and clinical sciences (Anatomy, Physiology, Pathology, Microbiology, Pharmacology and an introduction to clinical concepts), patient – doctor theme (the patient doctor relationship), community – doctor theme (the social, political, community and public dimensions of health) and the personal and professional development theme (bioethics and evidence-based decision making). In the second PBL session (PBL 2) students are provided with additional patient data and are expected to analyse this information on their own. In the third PBL session (PBL 3) the students, together with the facilitator, analyse all the learning material and provide explanations for the problem and suggest a plan of management for the problem. The PBL process is supported by a series of lectures and practical sessions. Once a week, “health practice days” provide the opportunity for early clinical exposure at the central teaching hospitals, instruction in the clinical skills laboratories and community engagement through service learning projects at schools and clinics.

The final two years of the programme (usually referred to as GEMP 3 and 4) are made up by clinical clerkships in the major disciplines such as internal medicine, surgery, psychiatry, paediatrics, obstetrics and gynaecology or combinations of smaller disciplines such as anaesthesiology, trauma, emergency medicine and public health. At the end of each of these clerkships, students are assessed in a variety of formats – the consequence of failing these assessments is that students may be expected to repeat that clerkship immediately which results in a delayed graduation. These summative assessments are made up of ward marks (a reflection of their performance in the clinical environment), written examinations and a variety of performance assessments such as the OSCE.

In 2010, the transformed curriculum delivered its fifth cohort of graduates into the South African health system. The aim of this study was to explore the perceptions of this group of graduates of the curriculum as they had experienced it at the point of graduation from the programme through the use of exit interviews.

## Methods

We undertook a qualitative descriptive and contextual study to explore graduating students’ perceptions about the programme. At their final examination session students were asked to complete a form if they were willing to participate in in-depth interviews or focus group discussions. Those who volunteered to be interviewed were contacted and placed into single interviews, dyads or focus groups according to their preferences for date and time of meeting. This allowed us to triangulate our data collection and meant that participants could choose a setting in which they felt most comfortable to express their views. There were seven interviewers who all used the same set of questions. We addressed four main questions in the interviews. These were to identify the best and worst experiences during the programme, to describe experiences related to assessment, to reflect on preparedness for the clinical years of study and finally to reflect on changes participants might make to the programme. Consistent with qualitative methodology, the first two questions were broad open-ended questions that gave participants opportunity to become comfortable in the interview process and at the same time allowed them to reflect on all the years of their study. The question related to assessment was chosen because of the general acceptance that assessment drives learning [12] and the last question because students often see the programme as having two theoretical years followed by two clinical years. The interviews took place in the medical school building in spaces where privacy could be ensured, such as unused offices. Participants were asked to choose a pseudonym for the single interviews and in the case of dyads and focus group discussions, students were asked to choose a number by which they and the interviewer referred to one another. The interviews were audio-taped, with the participants’ permission. They were informed that the information generated from the interviews would be used for curriculum evaluation and development and possible publication and that approval had been obtained from the Human Research Ethics Committee of the University.

The taped interviews were transcribed verbatim and were checked for correctness by three of the interviewers who read the transcripts whilst listening to the audio-tapes. Content analysis of the transcripts was then undertaken using Tesch’s eight steps [13]. The authors all read the transcripts. We met on a weekly basis over several weeks and discussed each transcript, identifying concepts. Common concepts were then grouped and themes identified. The constant comparative method of analysis was not possible because the interviews were conducted within a short time span of one week. However, data saturation was achieved.

## Results

A total of four single interviews, one dyad and five focus group discussions were held with participants. There were a total of 35 participants with 23 being school entrants and 12 graduate entrants. Three focus groups were equally balanced with the same number of school entrants as graduates and one focus group was comprised only of school leavers. (See Table 1 for the demographic characteristics of the interview groups). Six themes were identified. These were: two separate programmes; problem-based learning and ‘Garmins’®; see patients for real, being seen as doctors; assessment: of mice and MCQ’s; a cry for support; personal growth and pride.

**Table 1:** Demographic Characteristics of Participants and Type of Interview

**Type of interview**	**Characteristics of participants (n = 35)**	
	**Graduate Entrants (n = 12)**	**School Entrants (n = 23)**
4 Single interviews	1 male	3 female
1 Paired Interview	1 female	1 male
5 Focus groups	1 male and 2 females	2 male
		4 female
	2 males and 2 females	1 male and 3 females
	3 male	1 male and 2 females
	2 male and 1 female	1 male and 2 female

### Two separate programmes

Participants made several comments about the structure of the curriculum and the integration of learning. While there were a number of positive comments about the structure of the curriculum, often the comments suggested that participants did not see the relevance of information until later. One student reflected:

"*“I would go back to my files and I’d think, … we actually did this.”*"

In contrast some participants felt that:

"“o*ur theory was great, we were complete clinical idiots.We had a lot of knowledge but we had no idea how to apply that knowledge …* (in the clinical situation).”"

Commenting on the relationship between GEMP 1 and 2 and preparation for GEMP 3 and 4, a participant noted that *“it links up perfectly.”* In GEMP I and 2 students are taught to master a systematic approach to examining patients before contact with real patients. This is a practical skill that is taught in the clinical skills unit. This opinion was not held by all the participants. One participant described the programme as follows:

"*“(the) GEMP is almost sort of two separate programmes …..where 3*^*rd*^*and 4*^*th*^*years (*ie GEMP 1 and 2) *are very different from 5*^*th*^*and 6*^*th*^*years (GEMP 3 and 4)*.”"

Participants felt that a great deal of information is covered in GEMP 1 and 2 but the relevance of the information is not apparent unless it is *“actually applied to real patients.”* The lack of application was particularly felt in relation to the learning of pharmacology. One participant described the benefit of learning pharmacology in the clinical situation, saying:

"*“where you (get to see) where you can use a certain medication … instead of … rote learning.”*"

The value of the clinical years was frequently referred to with one participant reflecting that the four years should be merged. S/he described it as follows,

"“*so* we *like study it and then do the prac, study it and then do the prac.”*"

There was unanimous agreement that participants felt a need for changes in the teaching of pharmacology and microbiology. Likewise, there was consensus across all the participant groups that the clinical experiences obtained at the different hospitals varied greatly. When participants had had the opportunity to be allocated to different hospitals at different stages in the programme it was described “*as the perfect combination.”* For some participants the difference in learning opportunities and standards was problematic.

"*“I think the biggest flaw with GEMP 3 and 4 is that there’s sometimes a very big discrepancy between different hospitals. I’ve never managed an MI (myocardial infarct,) I’ve never managed unstable angina. That to me is a problem.”*"

### Problem-based learning and ‘Garmins’ ®

Participants could see the value in problem-based learning (PBL) but felt that it was not contributing to their learning as it was intended.

"“O*ften the theory and the actual practice of what is meant to be happening doesn’t always come through as strongly as possibly it was meant to when it was theoretically put together.”*"

There was agreement that the PBL 2 session is not meeting its objective. One participant said:

"“*to me the PBL 2 was a waste of time. It’s an hour out of the programme that you can rather use to discuss maybe that week surrounding pharmacological agents or treatments because … in a session where you’re forced to sit and forced to act and contribute, you start thinking about things.”*"

There was acknowledgement that the group process contributed to the PBL process, as one participant stated “*we all know how groups can be …*,” while another participant stated:

"“*the way that PBLs are structured. It’s that you’re put in a group with all different types of people who you don’t know so you all get to interact and you get to learn life skills. And you go on to your clinical years put into different groups and you sort of develop that interaction with different kinds of people with different socioeconomic group.”*"

A positive group experience resulted in one student saying,

"“*I loved PBL … I really enjoyed PBL…I had some really good people in my group. I learnt more in PBL with those students than I did probably in lectures.”*"

There was consensus that facilitators influence the outcomes of PBL, with clinicians seen as being more comfortable with the subject matter as compared with non-clinicians. At the same time clinicians were seen as being more capable of guiding students in their learning. One participant described a good facilitator as a Garmin® (a navigation device):

"*“I would just look in a textbook… it was like I needed a Garmin® to find what I needed in a textbook..*. *a physician or a doctor you know,… they understand where you need to go, they can kind of be your Garmin® … And you really, really need that .”*"

### See patients for real, being seen as doctors

Despite the variability of the clinical experience where the participants often have to take responsibility for their own learning, the participants placed great value in their clinical teaching as bringing them successfully to a point where

"“*we’re walking out from almost working as interns to working as interns.”*"

Reflecting on the value of the clinical learning experiences, another participant stated:

"*“It is only in 5*^*th*^*and 6*^*th*^*year (GEMP 3 and 4) that I felt I learnt the most. That is when I became competent. I don’t think I’m completely competent yet, but I feel confident at the moment. And I think that is because of the clinical exposure I’ve had.”*"

There was a mixed response to the early clinical exposure with some participants appreciating the “*systematic approach which gets drilled into us in GEMP 1 and 2”* in the clinical skills unit. However, participants felt that this learning experience should be

"*“taken more seriously and more emphasis placed on(it)....you have to be here and you have to do it – and it must also be taught properly.”*"

This statement reflects the need for joint responsibility between teachers and learners in this process.

Participants commented on the dearth of good clinical teachers while expressing great praise for those teachers who excel.

"“*I really don’t think all of the guys are great at teaching but some of them were fantastic. There are some guys who really are good at clinical teaching and bringing concepts alive, you know exactly what’s going on when you walk out. And those guys are great.”*"

While participants appreciated the biopsychosocial approach to patient care, they reflected that in the earlier years it was a

"“*lost opportunity* because *you only see the value of it (the biopsychosocial approach) in GEMP 3 and 4 and at that point it’s in the past and you don’t really care about it anymore.”*"

This discussion was taken further when participants elaborated on the value of the clinical years:

"*“(I) honestly think that’s where you are made into a doctor;… how you are approaching a patient and that initial approach is really the crux of what makes a doctor and we only learnt that in 3 and 4 (fifth and sixth years); so I would definitely extend that to the maximum point possible.”*"

### Assessment: of mice and MCQ’s

Participants raised many issues in relation to assessment. The subjectivity of assessors and a lack of standardization in the assessment process in GEMP 3 and 4 were recurring comments as exemplified by the participant who said:

"“*it’s not about what you know … a lot of the time it’s about who examines you. … If you get a nice examiner … and if you get a nice patient… so if you get something straight forward like cholecystitis, you’ll pass that case.”*"

A contrasting but less common opinion was that:

"“*it’s (assessment) very well standardized because they have a marking sheet.”*"

These reflections on differences were related to the differing pass rates in the different disciplines.

The difficulty of questions in relation to the purpose of the assessment was interrogated, as one participant explained:

"*“the exam is not there to prove what you do know, but maybe to nail you for what you don’t. If you don’t know the management of lupus nephritis, what’s the tragedy as a GP (general practitioner)?”*"

Frequent mention was made of being assessed on rare conditions such as

"*“a polycystic kidney, … an asperger, … a Takayasus.”*"

In this regard there was a common perception that

"“*some people have better luck with those cases than others.”*"

The multiple choice question format was singled out as being:

"“*very random (*with the result that if) *the professors are gonna show me how clever they are, I’m just gonna throw darts at a multiple choice sheet.”*"

Subjectivity of assessors was particularly evident in the allocation of ward marks. One participant stated that:

"“*you either get the consultant who just doesn’t care and gives everyone a 90 or 100% … on the other extreme you get the consultant who uses the ward mark as their opportunity to nail students unfairly.”*"

Experiences of subjectivity in relation to ethnicity were also raised, as forms of both negative and positive discrimination. One participant felt that:

"“*everyone is aware of race, they either tend to be more liberal, but then on the(other) hand, there is a prejudice coming in. I know one of the guys in my rotation just gets nailed every single time for assessment, whereas he’s actually a very good student.”*"

Another participant described how he had adopted coping strategies to counteract examiner bias by learning

"“*very quickly that you go to the exam like a little mouse, avoid having a personality in the exam.”*"

Inadequate feedback was a problem both for those who did well as well as for those who had failed a rotation. As one participant said:

"“*there’s no value in me repeating a block without knowing why or how you are failed.”*"

Another stated:

"*“If you really want to grow you should know what is right and what is wrong.”*"

Clear insights into some of the problem areas in assessment were however often accompanied by recognition of an alignment between the learning and assessment. One participant stated:

"“O*verall generally our assessments are good because we’re covering good academic knowledge or theoretical knowledge with our MCQs and a little bit with our OSCEs. We’re covering good clinical skills assessments with the Clinicals or OSPEs, so I really do think it is actually well matched.”*"

### A cry for support

Participants described the impact which the clinical years had on them emotionally. In the words of one participant:

"“*in medicine (at a large academic hospital) there are 60 beds in (a) ward and people are sick . People don’t get exposed to that at our age, you know. You grow up quickly and if you don’t, you just suffer.”*"

Another student stated that:

"“*there are two different sufferings, there is the academic stressful situations and then the emotional things.”*"

The academic “sufferings” related to seeing colleagues fail and having to repeat a block of study while the “emotional things” related to “*seeing your patients pass away.”* In one of the focus group discussions participants described the impact that a counselling course had had on them personally.

"“*We got to sit in a group and the amount of people who opened up and said that things were bothering them and one said that you begin to think that is normal. It made a huge difference … when you start doing the psychosocial side of things, you can’t just teach us a lecture (sic), then throw us out there.”*"

### Personal growth and pride

Participants reflected on their personal growth with a sense of pride.

"“*The person I am now compared to who I was at the beginning of the fifth year are miles of difference. It was ridiculous, I was like a child, I wore long sleeves …so scared to come near anyone…now you just get in there, you know, you need that confidence.”*"

The value of developing skills in life long learning and evidence-based medicine was recognised through statements such as:

"“*the latest thing on treating congestive heart failure is this*, *and we have got to start getting into the idea of not all studies are great*.”"

Patient centredness was described as something that has to be learnt practically and as one of the concepts that distinguishes the programme from those at other universities. One participant explained it as follows:

"“*I think PD (patient-doctor theme) - it’s very underestimated in the course, it’s very important…that’s what differentiates us from other doctors.”*"

There was a clear recognition that having had a lecture about ethics or patient-centeredness does not ensure that the student will behave or act in such a manner. One participant explained:

"“*we are taught ethics and morals but I think there’s sometimes that …no okay we taught the students about ethics, they are now ethical…but that does not happen…for some.”*"

A need for a practical approach in the teaching of ethics particularly to prepare them for the world of work was expressed as follows:

"“*speaking of ethics*…*I don’t feel like we actually get taught the kind of ethics that we need out there to protect ourselves legally…you should be read practical cases.”*"

Participants reflected on a professional responsibility to their peers in the learning sites as they provided support and guidance:

"“*this year the fifth years came to us and we helped them....They asked, can you show us your patients…then they felt more confident.”*"

Community engagement was acknowledged as one of the rewarding experiences of the GEMP programme:

"*“going out into the community to educate the community and ja, give back as a medical student and help in the clinics*.”"

Several of the interviews concluded with an emotional statement, such as:

"*“It (the programme) pushed me to really do my best; as an aside I’d like to add that I think we’re all proud that we came to Wits. I know we complain, it’s the nature of beast, 200 students are not going to be happy. We are glad we came here…We’re ‘Proud Witsies*’.”"

## Discussion

The participants in our study spoke openly and honestly about the programme. Their experiences of the programme were expressed with enthusiasm and emotion. The considered remarks about the educational process and what constitutes “best practice” and what does not were worthy. The value of obtaining graduates’ opinions of the curriculum has been described in the literature [14]. Using a structured questionnaire, Jalili et al. [14] found that the majority of their graduates felt that the basic science courses lacked clinical relevance and did not rate their clinical clerkships favourably. Our participants had a similar view of the basic sciences but provided rich descriptions of the value of their clinical clerkships.

This group of graduating students highlighted the central role that clinical teaching played in their growing competence to work as interns. Their comments confirm Spencer’s [15] view that clinical teaching is at “the heart of medical education”. The variability in teaching which our participants described across teaching sites is a feature of other clinical teaching environments. Stark[16] describes the need for a partnership between teachers and learners in the clinical area. Her work has identified the different perceptions that consultants and students have of appropriate teaching and this may account for some of our participants’ views that some teachers *are not great at teaching.* This suggests the need for training clinical teachers to take teaching to a level above the exchange of facts [15]. Our participants described good clinical teachers as being able to bring *concepts alive.* Feelings of incompetency in clinical skills at the start of the clinical years expressed by participants is consistent with the findings of Eyal and Cohen [17]. In their study 74% of the participants felt that they had not had sufficient clinical simulations and 40% felt that the curriculum had not provided opportunities of clinical relevance. In our study this was frequently commented upon in relation to the participants’ understanding of “core knowledge”.

One area of concern is their reported lack of pharmacology teaching as regards this core knowledge. Our participants’ need for extra teaching in pharmacology is consistent with Watmough et al’s study [8]. As in our study, their participants wanted this teaching to be more “structured” such as in having dedicated pharmacology lectures as opposed to an integrative style of teaching the content which is an integral part of a spiral curriculum.

It appears that our participants report an increased acknowledgement of the value of the theory and clinical learning from their earlier years of study when they reach the latter parts of the degree. This is intended in a curriculum which spirals through the degree.

From the themes identified it becomes evident that the spiralling nature of the curriculum was not always obvious to the participants; that the clinical years of the curriculum were viewed as the most relevant, although there are associated problems such as a lack of standardization across hospitals; that there is considerable variation in the value of PBL; that the perceived lack of standardization in assessment needs attention and that student support structures need to be strengthened.

In our study, participants called for a restructuring of the second session of the PBL process, with many seeing value in the other two sessions. It may be that the information supplied to our students in this session is too complex for their level of study. The results of tests done on a patient population with complex and often multiple pathologies may require more guidance from teachers and reflects in the words of the participant who stated that “*I needed a Garmin® to find what I needed in a textbook..*. *a physician or a doctor.”* The use of subject experts versus non-content experts has been extensively researched. In reviewing several studies on the subject, Dolmans et al. [18] concluded that the findings are ambiguous. It is however, acknowledged that content experts tend to lead the group into teacher-directed activities. In a systematic review of 13 papers, Koh et al. [19] found that there was strong evidence that PBL promoted teamwork skills and the social and emotional aspects of health care. Our participants made frequent mention of the patient-doctor theme in their discussions, recognising that this is best learnt in the clinical setting. At the same time they were aware of the impact of the psychosocial approach to health care on their own emotions.

The recurring perception of subjectivity of the assessors and the lack of standardisation in assessments raises important concerns. Downing [20] emphasises the importance of intra- and inter-rater consistency in assessment of ward and clinical performance. Compromising on reliability has important implications for the resultant validity of the inferences of competence [21-23] and our findings suggest that steps must be taken to limit possible threats to validity as described by Downing and Haladyna [24]. More effective feedback to students following assessments is also indicated to improve future performance and enhance motivation. Burch et al. [25] describe how feedback after bedside formative assessment in undergraduate clinical clerkships heightens students’ insights into their own competence, impacting on learning behaviour and clinical reasoning. Gordon [26] is of the view that teachers who have the ability to reflect on feedback of their teaching play an important part in students’ personal and professional development.

Participants cries for support structures may be addressed by informal support groups [27] and the development of coping skills. Participants in Bombeke et al’s study [28] suggested small discussion groups for personal development and well-being. It was felt that overwhelming experiences could also be discussed in this forum. Participants’ awareness that a formal lecture does not ensure ethical or patient-centred doctors is supported by Gordon [26] who writes that personal growth is “grounded in experience”. Bombeke et al’s study [28] describes the “doctor-as-person as a central phenomenon steering student’s patient-centredness.” They go on to state “that medical education needs student-centred teachers and supervisors to model not only patient-centredness, but also self-care and self-awareness. These comments have meaning for our study in the light of participants’ perceptions of assessors’ subjectivity and the lack of feedback in assessments.

The limitations of this study are that we, the interviewers, did not all probe in the same manner, with some probing more than others. In addition, participants volunteered to participate in the interview process and this may have resulted in some bias in the information obtained.

## Conclusions

In conclusion, the focus group discussions were lively and conversational, with participants often talking together as they shared experiences. Their similar suggestions meant that they frequently finished each others’ sentences. The participants’ comfort with the programme and willingness to talk about their experiences has meant that several of our perceptions of the programme have been confirmed and despite a lack in some knowledge bases being expressed, participants are exiting the programme confident and ready to begin their internships. We found exit interviews to be a valuable means of gathering information from students for curriculum reviews.

## Competing Interests

The authors declare that they have no competing interests.

## Authors’ contributions

L GT identified the need for this research and suggested the idea of conducting exit interviews with the graduating class of 2010; facilitated several of the focus group discussions; checked and corrected several transcripts of the transcribed interviews; participated in analysis of the transcripts and contributed to the writing of the manuscript. PMcI helped to refine the research question and methodology for the study; checked and corrected transcripts of the transcribed interviews; participated in analysis of the transcripts and contributed to the writing of the manuscript. DMM assisted in the interview process; participated in analysis of the transcribed interviews and contributed to the writing of the manuscript. NM-S assisted in the interview process; participated in the analysis of the transcribed interviews and contributed to the writing of the manuscript. SC assisted in the interview process; participated in the analysis of the transcribed interviews and contributed to the writing of the manuscript. TM submitted the ethics clearance application to the relevant committee; obtained the necessary consents from participants; participated in the interview process; checked and corrected several of the transcribed interviews and participated in analysis of the transcripts. All authors read and approved the final manuscript.

## Pre-publication history

The pre-publication history for this paper can be accessed here:

http://www.biomedcentral.com/1472-6920/12/49/prepub
